# Epiphyseal bone formation occurs via thyroid hormone regulation of chondrocyte to osteoblast transdifferentiation

**DOI:** 10.1038/s41598-017-11050-1

**Published:** 2017-09-05

**Authors:** Patrick Aghajanian, Weirong Xing, Shaohong Cheng, Subburaman Mohan

**Affiliations:** 1 Musculoskeletal Disease Center, Veterans Affairs Loma Linda Healthcare System, Loma Linda, California United States; 2 Department of Medicine, Loma Linda University, Loma Linda, California United States; 3 Department of Orthopedics, Loma Linda University, Loma Linda, California United States; 4 Department of Biochemistry, Loma Linda University, Loma Linda, California United States

## Abstract

Endochondral ossification in the diaphysis of long bones has been studied in-depth during fetal development but not postnatally in the epiphysis. Immunohistochemical studies revealed that Sox9 and Col2 expressing immature chondrocytes in the epiphysis transition into prehypertrophic and hypetrophic chondrocytes and finally into osteoblasts expressing Col1 and BSP during postnatal day 7–10, when serum levels of thyroid hormone (TH) rise. Lineage tracing using *Rosa-td tomato* ^*Col2-Cre-ERT2*^ mice treated with tamoxifen indicated that the same Col2 expressing chondrocytes expressed prehypertrophic, hypertrophic, and subsequently bone formation markers in a sequential manner in euthyroid but not hypothyroid mice, thus providing evidence that chondrocyte to osteoblast transdifferentiation is TH-dependent. Vascular invasion was apparent at the time of bone formation but not earlier. *In vitro* studies revealed that TH acting via TRα1 promoted expression of SHH while TRβ1 activation increased IHH but inhibited SHH expression. SHH promoted expression of markers of immature chondrocytes but inhibited chondrocyte hypertrophy while IHH promoted chondrocyte hypertrophy. Based on our data, we propose a model in which TH acting through TRα1 and TRβ1, respectively, fine tune levels of SHH and IHH and, thereby control the transit of proliferating immature chondrocytes into mature hypertrophic chondrocytes to become osteoblasts at the epiphysis.

## Introduction

Traditional endochondral ossification has long been believed to be the major method by which post-natal bone formation occurs in the epiphysis. In canonical endochondral ossification, the immature chondrocytes form a template and secrete a type II collagen (COL2)a1 matrix. Chondrocytes subsequently become hypertrophic and secrete collagen 10 (COL10)a1 and matrix metalloprotease 13 (MMP13), which serve to degrade the COL2 matrix^1–3^. The chondrocytes then become apoptotic and the tissue is vascularized. While this is occurring, the invading vasculature transports osteoclasts to remove cartilage and then mesenchymal stem cells (MSCs) differentiate into osteoblasts and finally into mature osteocytes. Ossification of the long bones occurs after two processes during defined time periods described as primary and secondary ossification. It is well established that primary ossification begins at embryonic day (E) 14.5–15.5, while secondary ossification typically occurs during early post-natal development^4, 5^. Recent studies regarding endochondral ossification have suggested different mechanisms for this process as it relates to primary ossification during gestation, but no in depth study of the secondary ossification mechanism exists. With regard to primary ossification, Ono *et al*. have suggested that a subset of chondrogenic mesenchymal precursors give rise to osteoblasts (OBs) and stromal cells, as well as a subset of osterix (OSX)^+^ transient mesenchymal cell precursors which generate OBs independent of the adult mesenchymal progenitor population^6^. Conversely, studies by the de Crombrugghe group suggest that OBs arising from chondrogenic tissues emerge from the dedifferentiation and redifferentiation of hypertrophic chondrocytes into OBs^7–9^. It follows that secondary ossification may involve a method of endochondral ossification similar to one of these mechanisms.

Thyroid hormone (TH) is known to play an important role in endochondral ossification. Thyroid hormones, triiodothyronine (T_3_) and thyroxine (T_4_), contribute to fracture healing, bone maintenance and development^10^. Previous studies have suggested that TH reaches peak levels during secondary ossification center (SOC) formation in multiple rodent species and in humans during late pre- and early post-natal development^11^. A study by our group, in particular, suggested that TH was required for the formation of the SOC, and further that formation was dependent on Indian hedgehog (IHH) and OSX signaling^11^. It is known that the SOC forms between post-natal day (P) 5–14, however, the exact timing at which the SOC forms is unknown, and therefore the mechanism by which endochondral ossification occurs in the SOC has not been elucidated.

In order to determine the role of TH and the mechanism by which SOC endochondral ossification occurs, we have performed a stringent time course analysis in the presence or absence of TH. This, coupled with markers of bone development, vascularization, apoptosis and proliferation allowed us to pinpoint the time at which secondary ossification begins. Further, lineage trace analyses initiating from COL2^+^ immature chondrocytes confirm the origin of many of the cells in the SOC that persist into adulthood. Our findings suggest that the initiation of endochondral ossification at SOCs is not under the control of canonical endochondral ossification processes, but occurs via the direct transdifferentiation of chondrocytes to osteoblasts.

## Results

### Tibia epiphyseal ossification begins in week old pups

**Figure 1:**
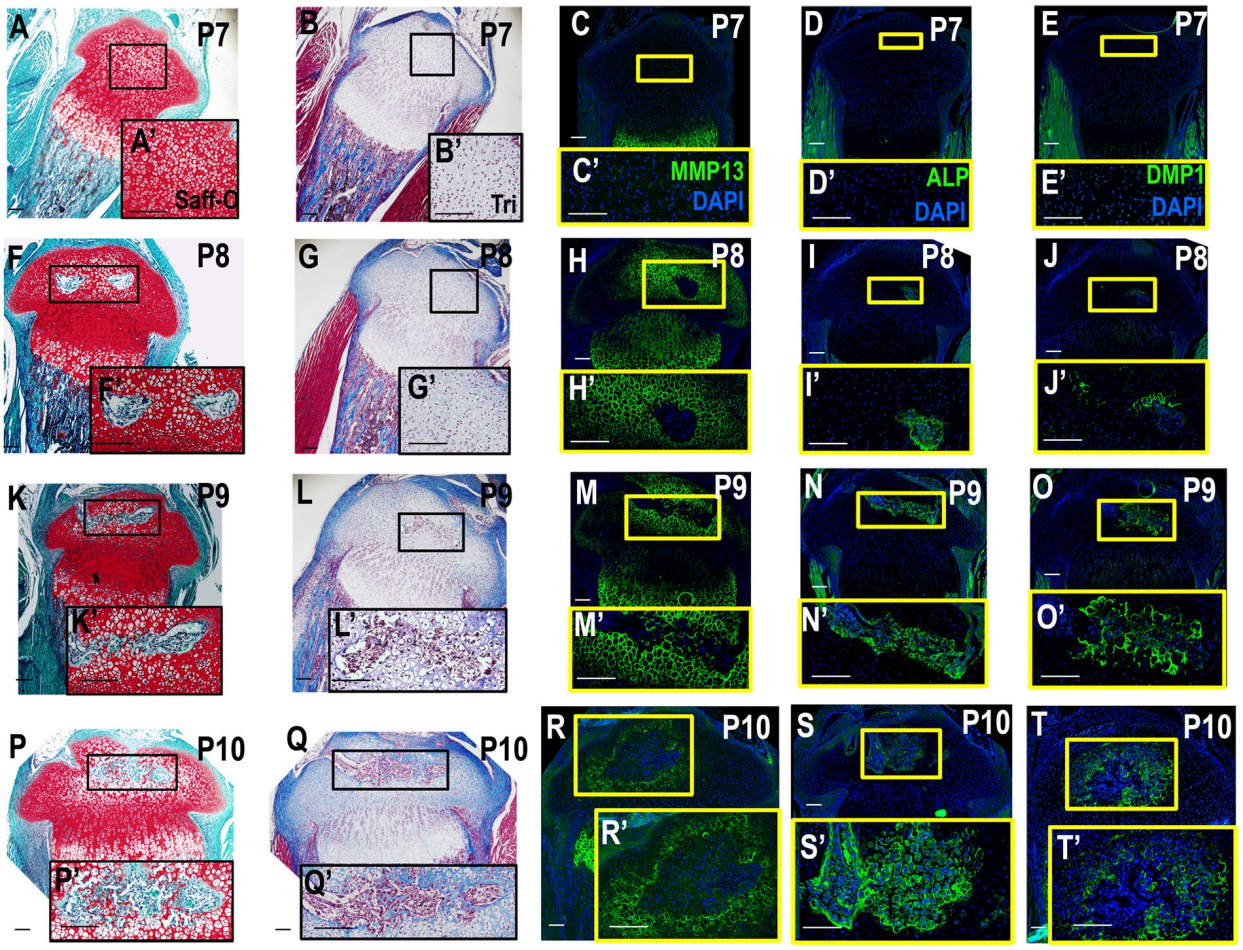
The SOC begins ossification at post-natal Day 8 as shown by osteogenic and hypertrophic markers. (**A**-**E**’) P7, no SOC ossification can be seen, however, hypertrophic chondrocyte markers begin expression in the center of the SOC as denoted by MMP13. (**F**-**J**’) Ossification begins in the center of the SOC at a late P8 time point, hypertrophic cells surround forming osteoblasts (**H**), an earlier P8 time point shows lack of osteoid as can be seen in trichrome stain samples (**G**). (**K**-**T**’) P9 and P10 show continued expansion of SOC ossification. Hypertrophic CCs surround the periphery of the expanding SOC (**M**,**R**). ALP (**N**,**S**) and DMP1 (**O**,**T**) enlarge expression in the SOC. Yellow or black boxes enlarge related regions, “ ’ ” denotes enlarged region (e.g., A’). Large Scale bars are 100 μm and small scale bars are 200 μm.

**Figure 2:**
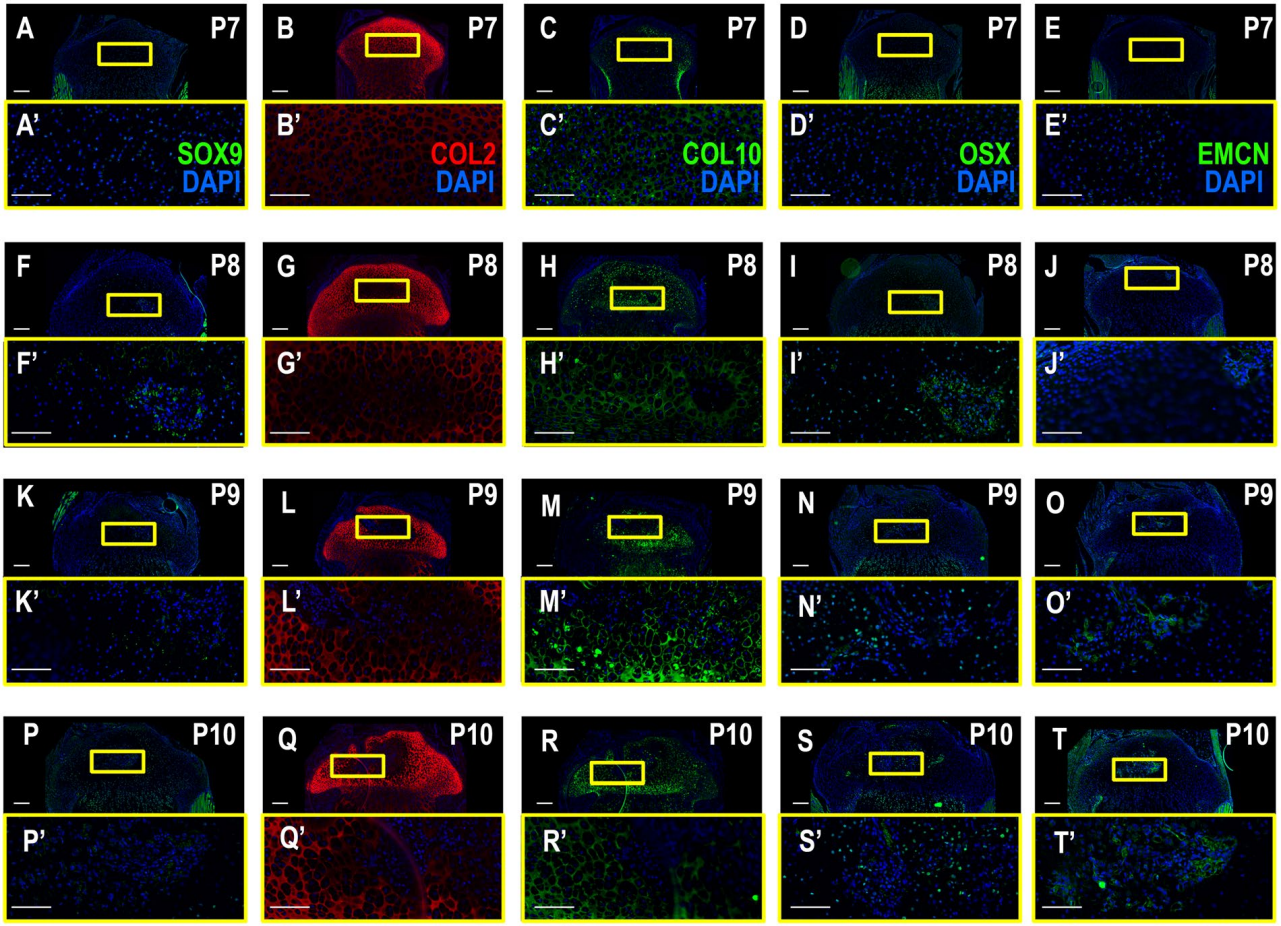
The SOC matures, but does not become vascularized until after ossification as shown by vascularization and immature, prehypertrophic and hypertrophic chondrocyte marker expression. (**A**–**E**’) P7, the SOC has not yet formed and the center of the epiphysis expresses immature and prehypertrophic CC markers (SOX9, COL2, COL10, OSX), but is not vascularized. (**F**–**J**’) P8, immature CC markers disperse from the center of the epiphysis, hypertrophic markers expand. Vascularization through EMCN can be seen in the invagination at the periphery of the epiphysis by the articular CCs (**J**). (**K**–**T**’) P9 and P10 SOX9, COL2 and COL10 expression disperse further from the central epiphysis. EMCN expression can be seen as invading blood vessels in the SOC starting at P9 (**N**,N’, **S**,S’). Yellow boxes enlarge related regions, “ ’ ” denotes enlarged region (e.g., A’). Large Scale bars are 100 μm and small scale bars are 200 μm.

**Figure 3:**
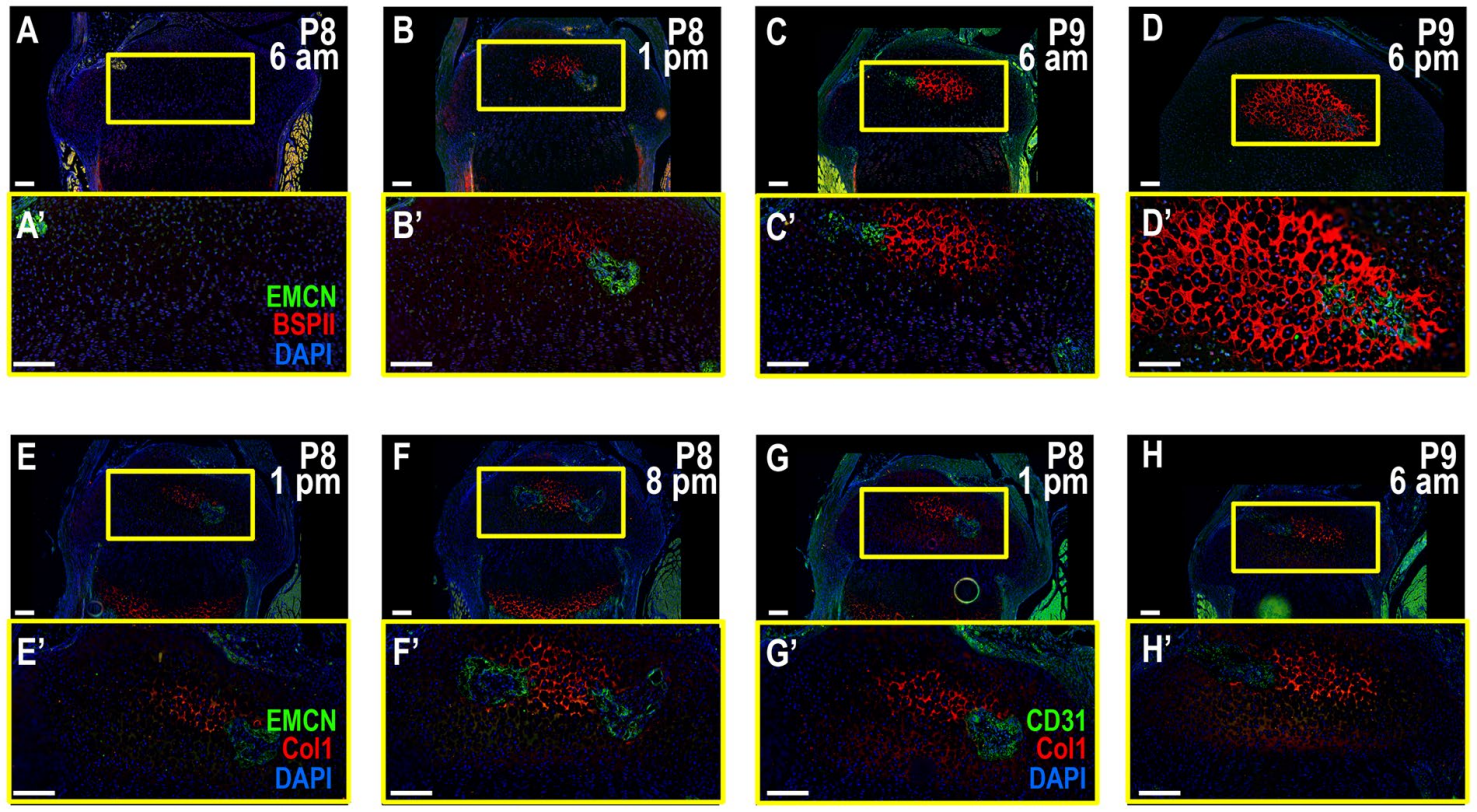
The bone begins to form prior to vascular invasion. (**A**-**D**) A time course analysis shows regions of bone formation denoted by BSPII (red) do not overlap with the invading vasculature visualized by EMCN (green) until the bone is already in the process of being formed. Time points indicate age and time of sacrifice. P8 6 am (**A**), P8 1 pm (**B**), P9 6 am (**C**), P9 6 pm (**D**). All sections are of proximal tibia except for (**D**) which displays the distal femur. (**E**,**F**) COL1 (red) co-stained with EMCN (green) shows a similar developmental pattern as the BSPII time course above. P8 1 pm (**E**) and P8 8 pm (**F**). (**G**,**H**) COL1 (red) co-stained with CD31 (green) indicates that the cells visualized by EMCN are indeed the invading vasculature. P8 1 pm (**G**) and P9 6 am (**H**). Yellow boxes enlarge related regions, “ ‘ ” denotes enlarged region (e.g., A’). “A” denotes significance. Large Scale bars are 100 μm and small scale bars are 200 μm.

We followed molecular and cellular events that occurred during initiation and progression of secondary ossification at the tibial epiphysis using an immunohistochemical time course analysis of the time points P6-P10. At P6-P7, chondrocytes in the region where the presumptive SOC will form do not display osteoid formation and do not express markers of hypertrophy or bone formation (Figs 1A–E and 2C,D, Fig. S1A–E,M,N), but do express high levels of SOX9 and COL2 (Figs 2A,B and S1K,L), markers expressed by immature chondrocytes. By P8, chondrocytes at the center of the epiphysis begin to express MMP13 and COL10, markers for hypertrophic chondrocytes (Figs 1H and 2H). However, SOX9 expression is reduced in the chondrocytes that undergo hypertrophy (Fig. 2F). Additionally, a small subset of cells in the center of the epiphysis and the prehypertrophic growth plate begin to express low levels of OSX (Fig. 2I). Between P8-P9, MMP13 and COL10 expression become segregated to the boundary around the forming SOC and the hypertrophic region of the growth plate (Figs 1H,M and 2H,M). Meanwhile, expression levels of COL2 and SOX9 are reduced and localized in epiphyseal chondrocytes outside the hypertrophic regions (Fig. 2F,G,K,L). At this time, cells also begin to express ALP and DMP1 (Fig. 1I,J) while continuing to express OSX, all markers of the osteogenic lineage, within the forming SOC. It is important to note that DMP1 and ALP expression begin late in the P8 period, as was confirmed in the 6 hour time course IHC analysis of the SOC (data not shown). This can be readily seen in samples stained with safranin-O which show the loss of chondrocytes in the epiphysis in the late P8 period (Fig. 1F), compared to trichrome stained sample during the early P8 period, which shows no osteoid formation (Fig. 1G). While vascular invasion was not apparent at P7 (Fig. 2E), we did observe a vascular invasion of the epiphysis using the endothelial marker endomucin (EMCN) beginning at the proximal edge of the epiphysis (Fig. 2J). It is important to note that the invasion of vascular tissue, while occurring concurrently with ossification, does not overlap with the SOC until P9 (Fig. 2O). This process was further examined in an hourly time-course analysis to view the exact impact of the vasculature on early bone formation via bone sialoprotein II (BSPII) (Fig. 3A–D) and Type 1 collagen (COL1)a1 with EMCN (Fig. 3E,F) and vascular marker CD31 (Fig. 3G,H). These analyses indicate that bone formation does, in fact, begin prior to vascular invasion of the center of the epiphysis. These vascular canals can also be observed in the P9 safranin O and trichrome stained samples (Fig. 1K,L), during which time osteoid layer formation can clearly be observed. Osteoid layer formation is not observed in earlier stage samples. Furthermore, the MSC marker, SCA1, was not observed in the forming SOC, but instead overlapped the location with invading blood vessels (Fig. S2C,F). By P10, the cartilage in the middle of the epiphysis was replaced by bone while the chondrocytes surrounding the newly forming bone began to express hypertrophic chondrocyte markers (Figs 1P,R and 2P–R) to expand the ossification peripherally towards the articular cartilage (Figs 1Q,S,T and 2S).

### Epiphyseal chondrocytes contribute to the bone formed in the SOC

**Figure 4:**
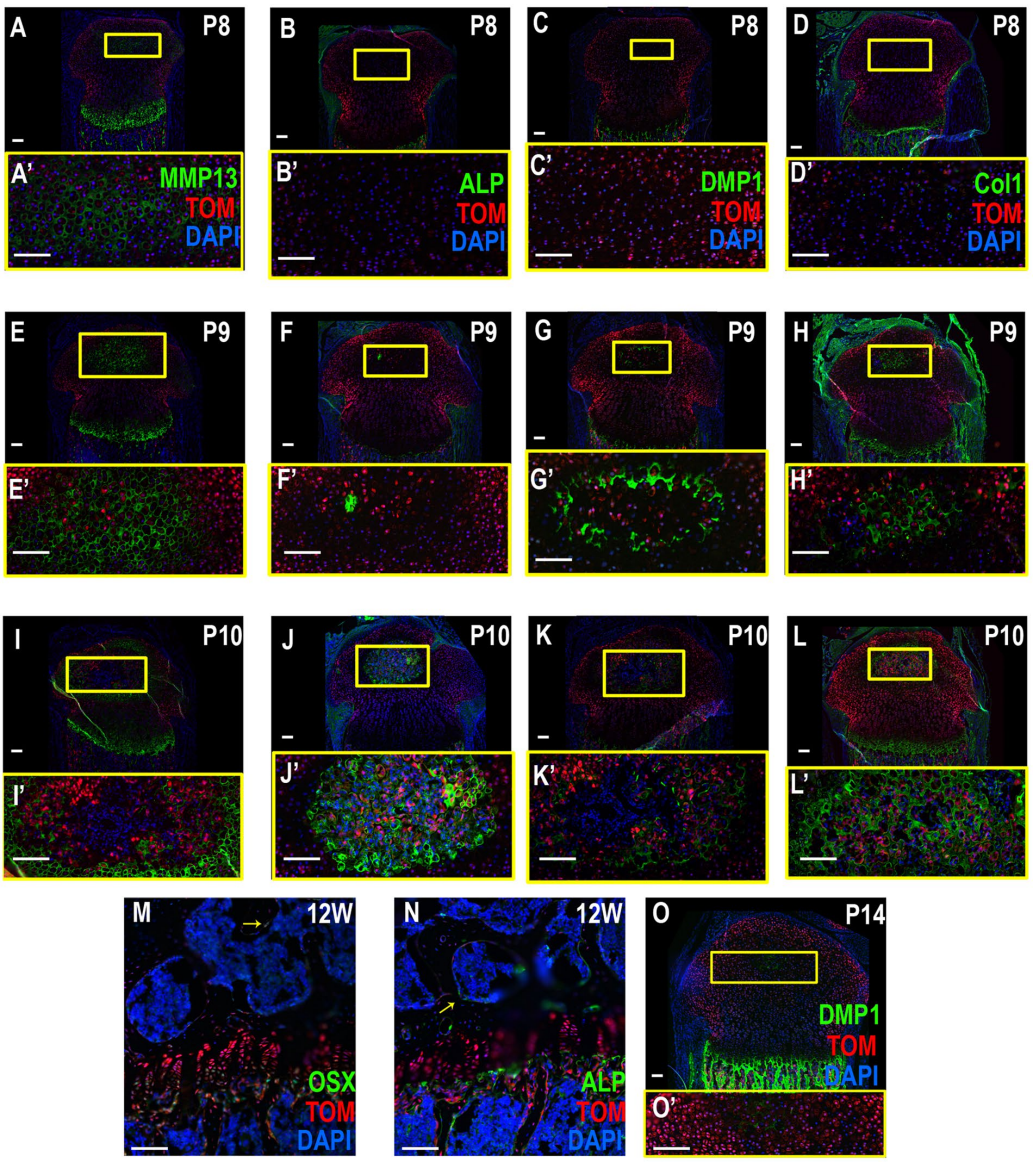
SOC osteogenic tissue arises from Col2 + chondrocytes (**A**–**I**’) Lineage trace analysis of *Col2-Cre* ^*ERT2*^ mice reveals that most cells in the epiphysis express TOM. As cells become hypertrophic (MMP13) (**A,E**) they continue to express TOM. Eventually, hypertrophic cells surround unlabeled TOM + cells (**I**). TOM + cells do, however, co-label with osteogenic markers such as ALP (**F**,**J**), DMP1 (**G**,**K**) and COL1 (**H**,**L**) at P9 and P10 during SOC formation. (**M**,**N**) Later stage, 12 week (12 W) *Col2-Cre* ^*ERT2*^ mice have SOCs which contain a large amount of TOM + cells as well as those colabeled with OSX (**M**; arrow) and ALP (**N**; arrow). (**O**) Methimazole treated *Col2-Cre* ^*ERT2*^ mice did not form an SOC and only expressed low levels of DMP1 by P14. The SOC is usually well into its formation at this time point. Yellow boxes enlarge related regions, “ ‘ ” denotes enlarged region (e.g., A’). Large Scale bars are 100 μm and small scale bars are 200 μm.

In order to determine the origin of the osteogenic tissue in the SOC, *Rosa-td tomato* ^*Col2-Cre-ERT2*^ mice were injected with tamoxifen at P3 and analyzed at days P6-P10. This allowed for lineage tracing from immature chondrocytes located in the epiphysis, but not from MSCs, which can also form osteogenic lineage cells. At P6, most cells in the epiphysis, except for those in the hypertrophic region of the growth plate, were COL2^+^ and SOX9^+^ (Fig. S1K,L), whereas all epiphyseal chondrocytes express tomato (TOM)^+^ reflecting COL2 promoter lineage expression. At P7, osteoid markers were not expressed; however Tom^+^ cells began to express MMP13 as they began to undergo hypertrophy (Fig. 4A,E,I). As noted before, the cells in the forming SOC became more specified toward the osteoblast lineage by day 8 and began to coexpress TOM and OSX (data not shown). This was followed by high coexpression of ALP, DMP1 and COL1 with TOM at P9–10 (Fig. 4F–H,J–L). This coexpression was sustained as the animal matured as seen in 12 (Fig. 4M,N) and 16 week old mice (data not shown). It is important to note, there were no detectible MSCs in the SOC until it became vascularized. Moreover, little to no apoptosis was observed in the forming SOC as confirmed by a lack of active caspase 3 (CAS3) staining of the TOM^+^ cells (Fig. S2A,B). Finally, to determine whether the osteoblasts of the SOC arose from a rapidly proliferating stem cell population, sections were stained with KI67. While the epiphysis did show high expression of KI67 in regions such as the growth plate, the presumptive SOC exhibited no KI67 signal (Fig. S2C,D). This suggests a different mechanism for endochondral ossification not previously identified. Thus, taken together, and given the limited period of time when ossification occurred in the SOC, the data suggests that osteoblasts of the SOC arise directly from the epiphyseal chondrocytes.

### TH directly induces chondrocyte transdifferentiation into osteoblastic lineage cells in the SOC

**Figure 5:**
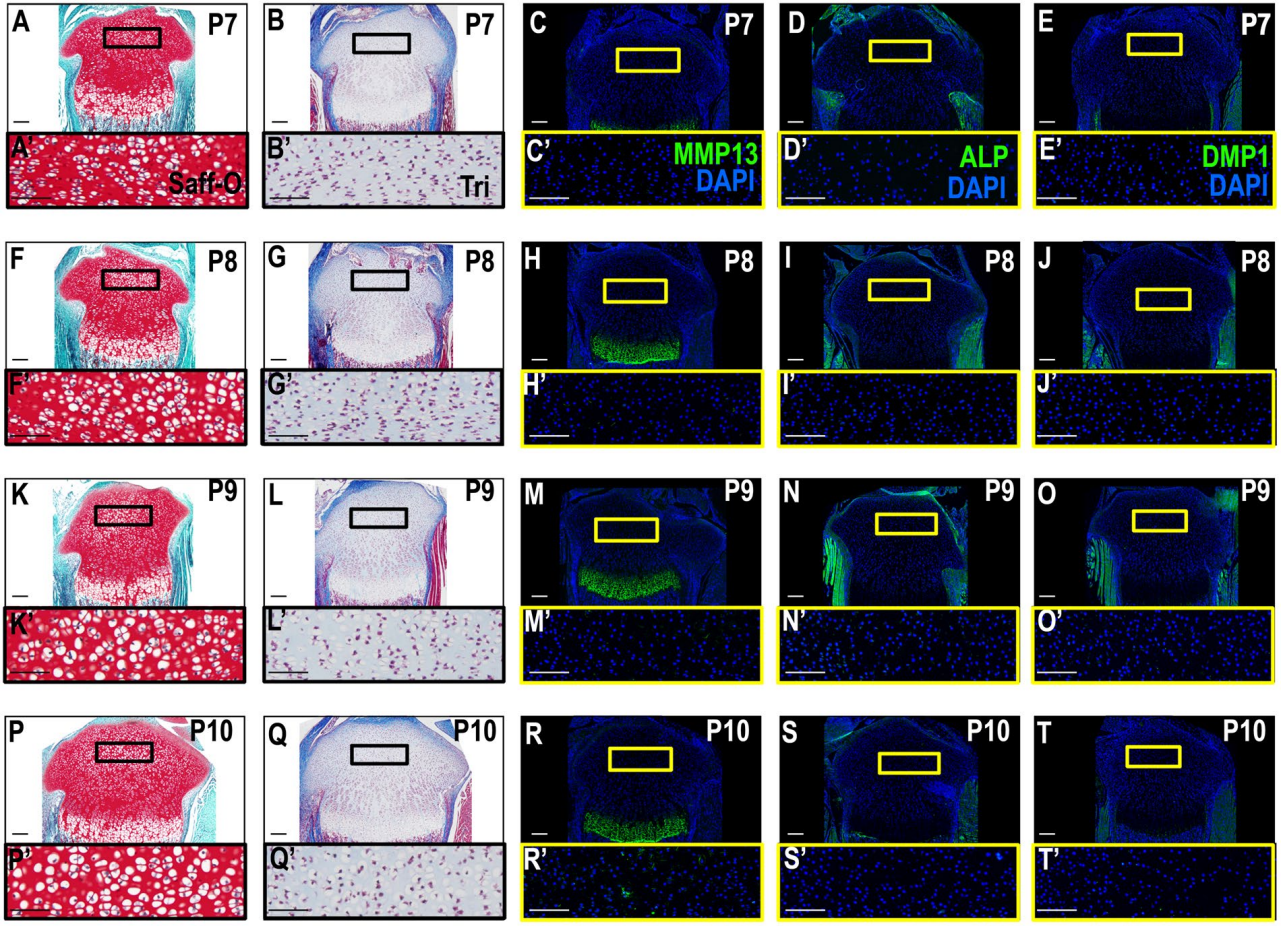
Thyroid hormone is required for CC hypertrophy and osteogenesis in the epiphysis. (**A**–**T**’) *Hyt/hyt* mice do not form an SOC between P7-P10 as confirmed by safranin-O (**A**,**F**,**K**,**P**), trichrome (**B**,**G**,**L**,**Q**), lack of CC hypertrophy via MMP13 (**C**, **H**, **M**, **R**), and lack of osteogenic expression via ALP (**D**,**I**,**N**,**S**) and DMP1 (**E**,**J**,**O**,**T**). Yellow or black boxes enlarge related regions, “ ’ ” denotes enlarged region (e.g., A’). Large Scale bars are 100 μm and small scale bars are 200 μm.

**Figure 6:**
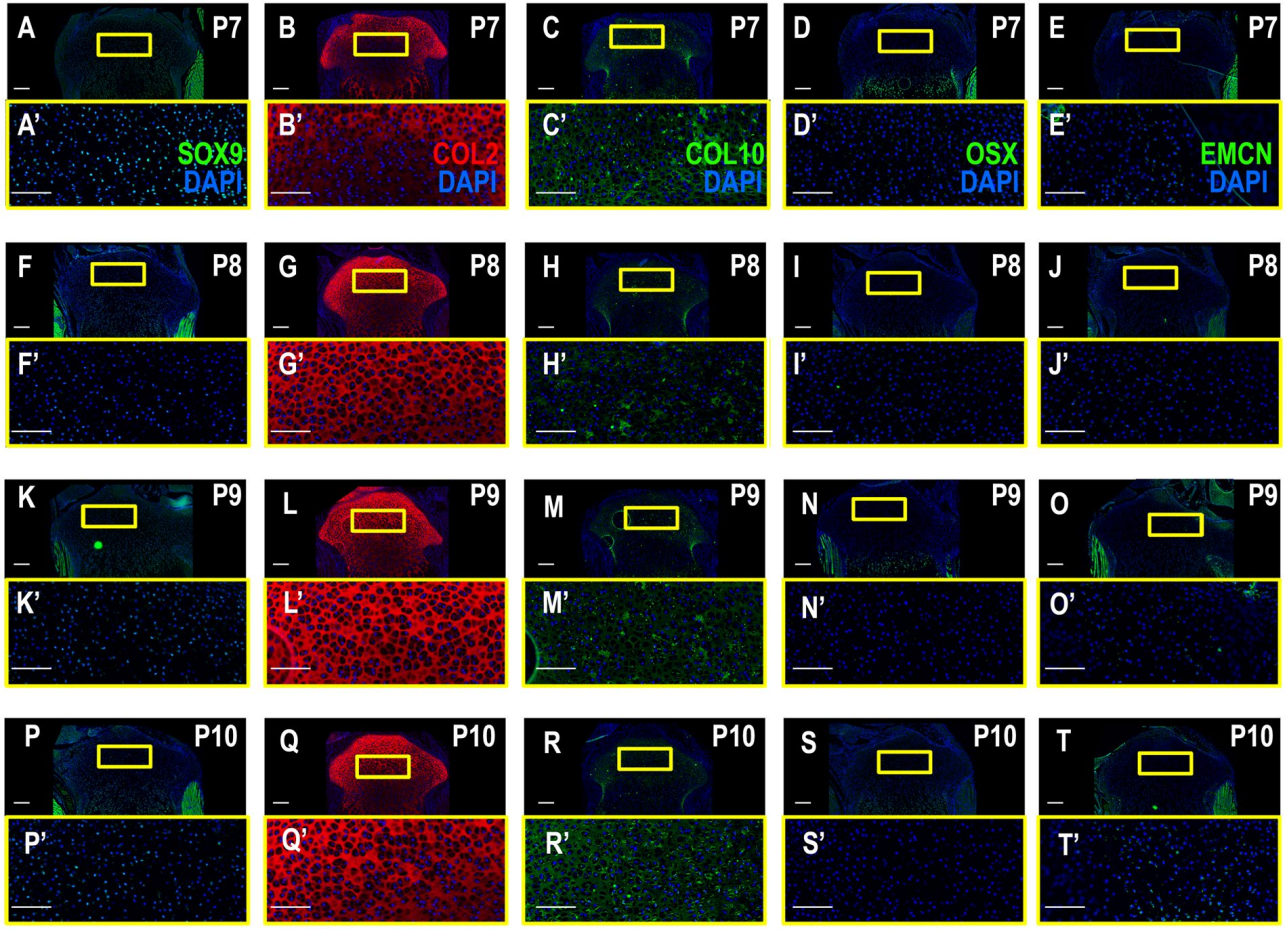
CCs do not mature and no vascularization occurs in the epiphysis in the absence of thyroid hormone. (**A**–**T**’) Between P7-P10 *hyt/hyt* mice do not form mature epiphyseal CCs as seen by high expression of SOX9 (**A**,**F**,**K**,**P**) and COL2 (**B**,**G**,**L**,**Q**), remained prehypertrophic via COL10 (**C**, **H**, **M**,**R**), and lack of osteogenic expression via OSX (**D**,**I**,**N**,**S**). Further, no vascular invasion was detected via EMCN (**E**,**J**,**O**,**T**). Yellow boxes enlarge related regions, “ ’ ” denotes enlarged region (e.g., A’). Large Scale bars are 100 μm and small scale bars are 200 μm.

It has previously been shown that the SOC forms within the time period that post-natal TH levels peak in mice (P5-P14), and that TH is required for normal SOC formation^11^. In order to identify the cellular mechanism by which TH influences this event, we performed a time course analysis of SOC formation with TH deficient *Tshr* ^*hyt/hyt*^ (*hyt/hyt*) animals (Figs 5 and 6, Fig. S1F–J,P–T). No SOC formation or markers of bone formation were observed between P6-P10 (Figs 5 and 6, Fig. 1F–J,P–T). The epiphyses of *hyt/hyt* animals did, however, express high levels of SOX9 and COL2 (Figs 6A,B,F,G,K,L,P,Q and S1P,Q), while also expressing low levels of COL10 (Figs 6C,H,M,R and S1R). Measurement of gene expression changes by real time PCR showed that while expression levels of osteoclast markers, cathepsinK (*CtsK*) and calcitonin receptor (*Ctr*), were unchanged in the hypothyroid *Tshr* ^*hyt/hyt*^ mice, expression levels of *Mmp13*, *AdamTS4* and *AdamTS5* were significantly reduced in the *Tshr* ^*hyt/hyt*^ mice (Fig. S3). Furthermore, to examine how the suggested transdifferentiation mechanism may be affected by reduced levels of TH, we supplemented the water of pregnant *Rosa-td tomato* ^*Col2-Cre-ERT2*^ mice with methimazole, which inhibits the synthesis of triiodothyronine (T_3_) and thyroxine (T_4_). As expected, treatment with methimazole nearly ablated the formation of the SOC in mice at P14. At this stage all cells in the epiphysis were TOM^+^. While OSX was coexpressed in many TOM^+^ cells in the presumptive SOC location, their expression levels were rather low as was also the case for DMP1. However, no expression of ALP was observed in the TOM^+^ chondrocytes at the middle of epiphysis at day 14 in these hypothyroid pups (Figs 4O and S4A,B). Conversely, chondrogenic markers such as COL2 and MMP13 were still widely expressed in the SOC (Fig. S4C,D). As noted earlier, vascular invasion was not observed in the epiphysis in *hyt/hyt* animals (Fig. 6E,J,O,T), yet TOM^+^ cells began to express prehypertrophic and osteogenic markers. This suggests that the transdifferentiation of the presumptive SOC chondrocytes to bone is dependent on TH status.

### SHH and IHH signal balance is required for chondrocyte transdifferentiation

**Figure 7:**
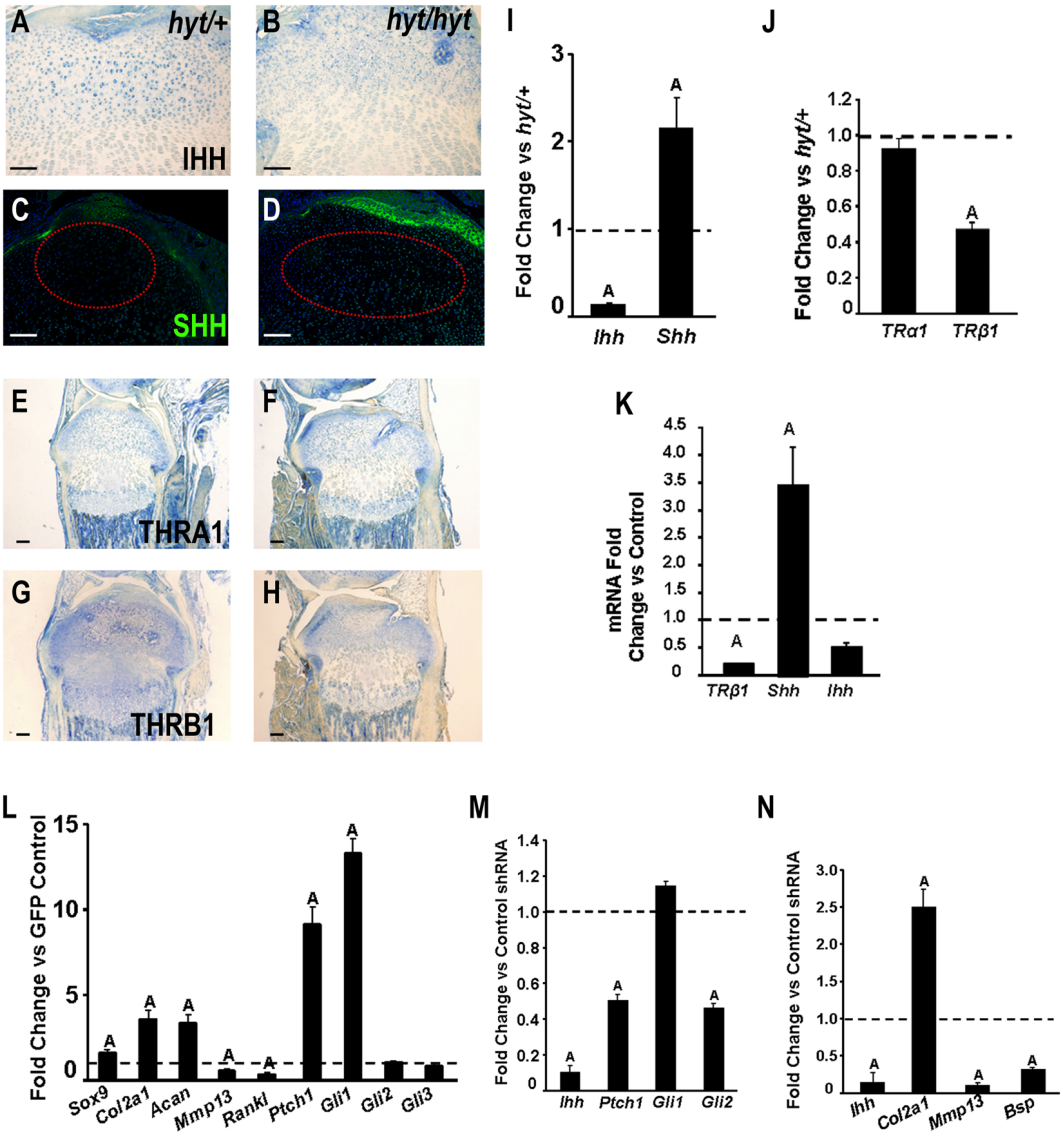
Thyroid hormone acts through THRβ1 and HH expression to influence epiphyseal chondrocyte differentiation. (**A**–**D**) IHC data confirms a higher IHH expression in the SOC vs SHH (green) in P7 *hyt/ + *mice (**A**,**C**), whereas there is an extreme reduction of IHH expression coupled with an increase in SHH expression in *hyt/hyt* mice (**B**,**D**). (**E**–**H**) IHC data indicate unchanged THRα1 levels in both *hyt/ + *and *hyt/hyt* animals (**E**,**F**). Conversely, THRβ1 expression is markedly reduced in *hyt/hyt* compared to *hyt/ + *animals (**G**,**H**). (**I**,**J**) *Ihh* mRNA expression of P7 epiphyses decreased 7 fold while *Shh* expression is increased 2.4 fold in *hyt/hyt* vs *hyt/ + *animals (**I**). P7 epiphyses of *hyt/hyt* animals showed a 2.2 fold decrease in *Thrβ1* but no effect in *Thrα1* expression compared to *hyt/* ^+^animals (**J**). (**K**) *Thrβ1* knockdown in cultured CCs increased *Shh* expression 3.4 fold and reduced *Ihh* expression 2 fold. (**L**) Adenoviral OE of *Shh* in epiphyseal CCs increased *Sox9, Col2*, and *Acan* mRNA expression (1.5, 3.5, and 3 fold, respectively). *Mmp13* and *Rankl* expression decrease 1.7 and 2.8 fold, respectively. *Ptch1* and *Gli1* expression is increased 9 and 13.5 fold, respectively. *Gli2* and *Gli3* expression remain unchanged. All mRNA is tested against GFP adenovirally treated cells. (M, N) shRNA knockdown of *Ihh* in ATDC5 CCs causes an increase in *Gli1*, however, *Ptch1* and *Gli2* are knocked down (2 fold each) (**M**) It also reduces *Mmp13* and *Bsp* expression (5 and 3.3 fold, respectively) but increases *Col2* expression (2.5 fold) (**N**). Red Circles denote the SOC initiation region. All RNA data are represented as a mean ± SEM, “A” denotes significance. Large Scale bars are 100 μm and small scale bars are 200 μm.

Because indian hedgehog (IHH) is known to act downstream of TH and THRβ1 in order to form the SOC^11, 12^, we examined hedgehog signaling in epiphyseal chondrocytes. IHC data confirmed a higher IHH expression in the SOC vs SHH in 7 day old euthyroid *hyt/* *+ *mice (Fig. 7A,C), whereas there was an extreme reduction of IHH expression coupled with an increase in SHH expression in *hyt/hyt* mice (Fig. 7B,D). While expression levels of THRα1 was unaffected by TH status in the epiphyseal chondrocytes at day 7 (Fig. 7E,F), THRβ1 expression was reduced in the absence of TH (Fig. 7G,H). Consistent with the immunohistochemistry data, Ihh mRNA expression was decreased 7 fold while *Shh* expression was increased 2.4 fold in *hyt/hyt* animals (Fig. 7I). While the mRNA level of *Thrα1* was not different between the two genotypes, expression levels of *Thrβ1* was reduced by 2.2 fold in the epiphysis of *Tshr* ^*hyt/hyt*^ mice at day 7 (Fig. 7J). This was further confirmed with treatment of *hyt/hyt* animals with T_3_/T_4_, which increased *Thrβ1* but not *Thrα1* expression in the SOC (data not shown). To understand the role of THRβ1 regulation of hedgehog signaling in regulating epiphyseal chondrocyte transdifferentiation, we performed multiple culture assays with primary epiphyseal chondrocytes. First, we assayed the effect of knocking down *Thrβ1* expression specifically with *Thrβ1* lentiviral shRNA treatment and measured *Shh* and *Ihh* expression levels. *Thrβ1* knockdown in cultured chondrocytes increased *Shh* expression ~3.4 fold but reduced *Ihh* expression 2 fold (Fig. 7K). *Shh* was then overexpressed in epiphyseal chondrocytes to examine its effect on the expression of various markers of chondrocyte maturity. Adenoviral overexpression of *Shh* in epiphyseal CCs promoted expression of immature chondrocyte markers *Sox9* (~1.5 fold), *Col2* (~3.5 fold) and aggrecan (*Acan)* (~3 fold), while it decreased expression of mature CC markers *Mmp13* (~1.7 fold) and *Rankl* (~2.8 fold) (Fig. 7L). Finally, we examined downstream effectors of hedgehog signaling. *Shh* overexpression increased expression of some, but not all downstream hedgehog effectors. *Ptch1* and *Gli1* increased (~ 9 fold and ~13.5 fold respectively) while *Gli2* and *Gli3* remained unchanged (Fig. 7L). Similarly, shRNA knockdown of *Ihh* in epiphyseal chondrocytes caused a decrease in *Ptch1* and *Gli2* expression levels (~2 fold each) but not in *Gli1* (Fig. 7M). Furthermore, knockdown of *Ihh* reduced expression of markers of chondrocytes (*Mmp13*) and osteoblasts (*Bsp*) (Fig. 7N). Taken together, these data suggest that SHH promotes an immature, stem like fate for chondrocytes via the specific mediator, GLI1. On the other hand, IHH promotes chondrocyte hypertrophy and osteoblast differentiation via the specific mediator, GLI2. TH acting via THRβ1 suppresses SHH but increases IHH expression.

## Discussion

In this study, we followed the cellular and molecular events that occurred during secondary center ossification of the epiphysis in euthyroid and hypothyroid mice to get an insight into the mechanisms for endochondral bone formation. Our focus on the epiphysis was based on the following: 1) Secondary center ossification at the epiphysis occurs quite rapidly in mice (postnatal days 7–10), thus providing a narrow window in time for mechanistic studies, and 2) Initiation and progression of secondary center ossification occurs at a time when serum levels of T_3_ increase in several species including humans, and hypothyroid mice exhibit a severe delay in secondary center ossification at the epiphysis^11, 13, 14^, thus providing a model for evaluation of mechanisms for TH regulation of endochondral bone formation. By studying endochondral bone formation at the epiphysis during the prepubertal growth period, we found an important role for hypertrophic chondrocytes to produce bone matrix through direct conversion to osteoblasts. Our data demonstrate that epiphyseal chondrocytes drive the entire sequence of events in the endochondral bone formation pathway, from removal of cartilaginous matrix to subsequent replacement with mineralized bone and that TH regulation of SHH/IHH signaling is critically involved in these processes.

The prevailing model for endochondral ossification dictates that proliferating chondrocytes mature into hypertrophic chondrocytes to produce mineralized cartilage and promote vascularization and remodeling through production of certain local growth factors. Hypertrophic chondrocytes are also known to direct adjacent perichondral cells to become osteoblasts to form the bone collar^15, 16^. Then, the hypertrophic chondrocytes progressively undergo apoptotic cell death and the discarded cartilage matrix provides a scaffold for osteoblasts to lay down true bone matrix. This model dictates that cells brought in through invading vasculature are the main source of osteoclast precursors to resorb cartilage and osteoblast precursors to form bone^17, 18^. However, recent studies from a number of laboratories including ours have supported an alternate paradigm for endochondral bone formation in which the hypertrophic chondrocytes are themselves the main source of bone forming osteoblasts^6–9, 19–21^.

At postnatal day 5, much of the epiphysis is occupied by immature chondrocytes that are positive for COL2 and SOX9. Our lineage tracking studies using a tomato red reporter construct expressed in chondrocytes demonstrate that the COL2 and SOX9 expressing chondrocytes that are marked red go on to express markers of prehypertrophic (IHH), hypertrophic (COL10 and MMP13) and osteoblasts (OSX, DMP1, ALP, COL1) progressively between postnatal day 7–9 (Fig. 4A–L). Furthermore, our data show that some of these Col2 expressing chondrocytes marked with tomato red on postnatal day 3 go on to become osteocytes and were seen in the trabecular bone of the epiphysis after several weeks (Fig. 4M,N). Our data as well as other recently published data show that transdifferentiation of hypertrophic chondrocytes into osteoblasts is not unique to secondary center ossification but also may be relevant during primary center ossification as well as during fracture healing^6, 7^.

Our immunohistochemistry data using various cell type-specific markers show that chondrocytes at the middle of epiphysis began to express Col10 but not MMP13 at postnatal day 6. However, at postnatal day 7, MMP13 expression was seen in the middle but not in the periphery of the epiphysis. At postnatal day 8 and 9, MMP13 expression was lost in the middle but persisted in the periphery of the epiphysis. Little or no expression of DMP1 and ALP were seen in the middle or in periphery of the epiphysis at postnatal day 6 or 7. However, at postnatal day 8, DMP1 and ALP1 expression was seen in the middle of the epiphysis which then extended in a circular fashion peripherally at P9 and P10. The expression of bone markers at the center of epiphysis was followed by mineralization at postnatal day 9. These data suggest that chondrocyte conversion from prehypertrophic to hypertrophic and then to osteoblasts occurred very rapidly within a matter of 2–3 days at the epiphysis during secondary center ossification.

It has been widely accepted that hypertrophic chondrocytes undergo apoptosis prior to the invasion of bone marrow-derived mesenchymal cells to provide a source of osteoblasts^17^. If this is true at the secondary center ossification, we would anticipate death of hypertrophic chondrocytes, followed by neoangiogenesis prior to expression of osteoblast specific markers at the epiphysis on postnatal day 8. If the invading vasculature is the major source of osteoblast precursors, as the original paradigm of endochondral bone formation suggest, then we would anticipate neoangiogenesis to occur prior to expression of osteoblast marker genes. Our immunohistochemistry data, using antibodies against an apoptotic marker, active-caspase 3, revealed no evidence of significant apoptosis of hypertrophic chondrocytes at postnatal day 7 (Fig. S2A,D). Furthermore, our data using angiogenic markers, EMCN and CD31, indicate that the SOC is already populated with osteogenic tissue prior to the blood vessels being formed (Figs 1, 2 and 3). The blood vessels do not transport osteoblasts, but instead bring in immature MSCs or osteoblastic precursors, which then must propagate and differentiate into osteoblasts^22^, thus requiring additional time. There was little evidence of cell proliferation using the Ki67 marker at the epiphysis where bone is being formed at postnatal day 8 or 10 (Fig. S2B,E). These data collectively argue against the established paradigm that invading vasculature is the main source of bone forming osteoblasts during endochondral ossification at the epiphysis.

While our data seem to be consistent with a model in which hypertrophic chondrocytes transdifferentiate into osteoblasts directly during secondary center ossification at the epiphysis, another study by Zhou *et al*.^7^ seems to favor the idea of hypertrophic chondrocytes dedifferentiating first prior to differentiation into osteoblasts. If this were true, we would predict the process of dedifferentiation of hypetrophic chondrocytes into precursors and their redifferentiation into osteoblasts would require additional time to occur, thus leading to the conclusion that, in order to begin bone formation within the stringent developmental temporal controls, the most logical explanation is the direct transdifferentiation of hypertrophic chondrocytes into osteoblasts. We would like to suggest further, that based on this, the hypertrophic chondrocytes themselves, are a misnomer, and they are, in fact, preosteoblasts. This may explain why factors such as OSX or DMP1 are expressed in these cells, at least in low levels. While our data provide strong evidence for hypertrophic chondrocytes as a major source of osteoblasts during secondary center ossification, the issue of whether bone marrow-derived MSCs also contribute significantly to the preosteoblast and osteoblast population and whether chondrocytes continue to contribute to endochondral bone formation throughout adulthood remains to be established.

In our studies to understand the molecular pathways that contribute to chondrocyte to osteoblast transdifferentiation during secondary center ossification, we focused on TH since the rise in serum levels of TH coincides with initiation and progression of secondary center ossification at the epiphysis and since treatment of hypothyroid mice with TH between postnatal days 5–14 completely rescued the trabecular bone formation deficit at the epiphysis^11, 12, 14^. Observations consistent with an important role for TH in chondrocyte to osteoblast transdifferentiation, COL2 expressing tomato red positive chondrocytes failed to express bone formation markers at postnatal day 14 in methimozole treated mice (Figs 4O and S4A,B). Furthermore, our immunohistochemistry data indicated that expression of IHH and OSX, known initiators of osteogenic fate determination, was severely compromised in the epiphyseal chondrocytes of TH deficient *hyt/hyt* mice (Figs 6D,I,N,S and 7B). Our data clearly show a loss of OSX expression in the epiphysis, but beyond the expression of OSX, cells of the epiphysis of *hyt/hyt* mice did not become hypertrophic, and instead remained immature chondrocytes throughout our time course experiments. This, however, was not true for the expression of hypertrophic markers in the growth plate or markers of bone in the trabecular bone, leading to the conclusion that TH must temporally activate these factors and initiate these developmental programs to promote chondrocyte to osteoblast transdifferentiation and bone formation specifically at the epiphysis.

TH actions in bone are known to be mediated via TH receptor (THR) α1 and THRβ1^23–26^. While THRα1 has been shown to be constitutively expressed at high levels in progenitor cells, proliferating chondrocytes and osteoblasts, THRβ1 expression was seen only in a subset of cells (prehypertrophic) and its expression is known to be induced by TH in a THRα1-dependent mechanism^27^. Our data show that the expression levels of THRβ1 in the epiphyseal chondrocytes was clearly reduced in the TH deficient *hyt/hyt* mice and that TH treatment promoted THRβ1 expression. THRα1 is essential for longitudinal bone growth and acts via promoting SHH expression^10, 28, 29^. SHH increases *Sox9*, *Sox5* and *Sox6* expression which subsequently stimulates expression of *Col2* and aggrecan and suppresses *Col10* expression. *Sox9* is important for proliferation and survival of chondrocytes^30^. Targeted expression of SHH in *Col2* expressing chondrocytes blocked chondrocyte differentiation at prehypertrophic stage, hindering endochondral ossification and trabecular bone formation^30^. By contrast, THRβ1 is essential for chondrocyte differentiation and acts via promoting IHH signaling^11, 12, 31^. We recently showed that ligand binding to THRβ1 induces expression of IHH and OSX and contributes to trabecular bone formation at the epiphysis^11, 12^. In addition, a recent study showed that IHH promoted chondrogenic differentiation into osteoblasts during adult zebrafish jawbone regeneration^20^. Thus, while SHH is essential for maintenance of undifferentiated chondrocyte populations by preventing their premature entry into a hypertrophic status, IHH signaling appears to be critically involved in promoting chondrocyte hypertrophy and osteoblast development during secondary center ossification.

**Figure 8:**
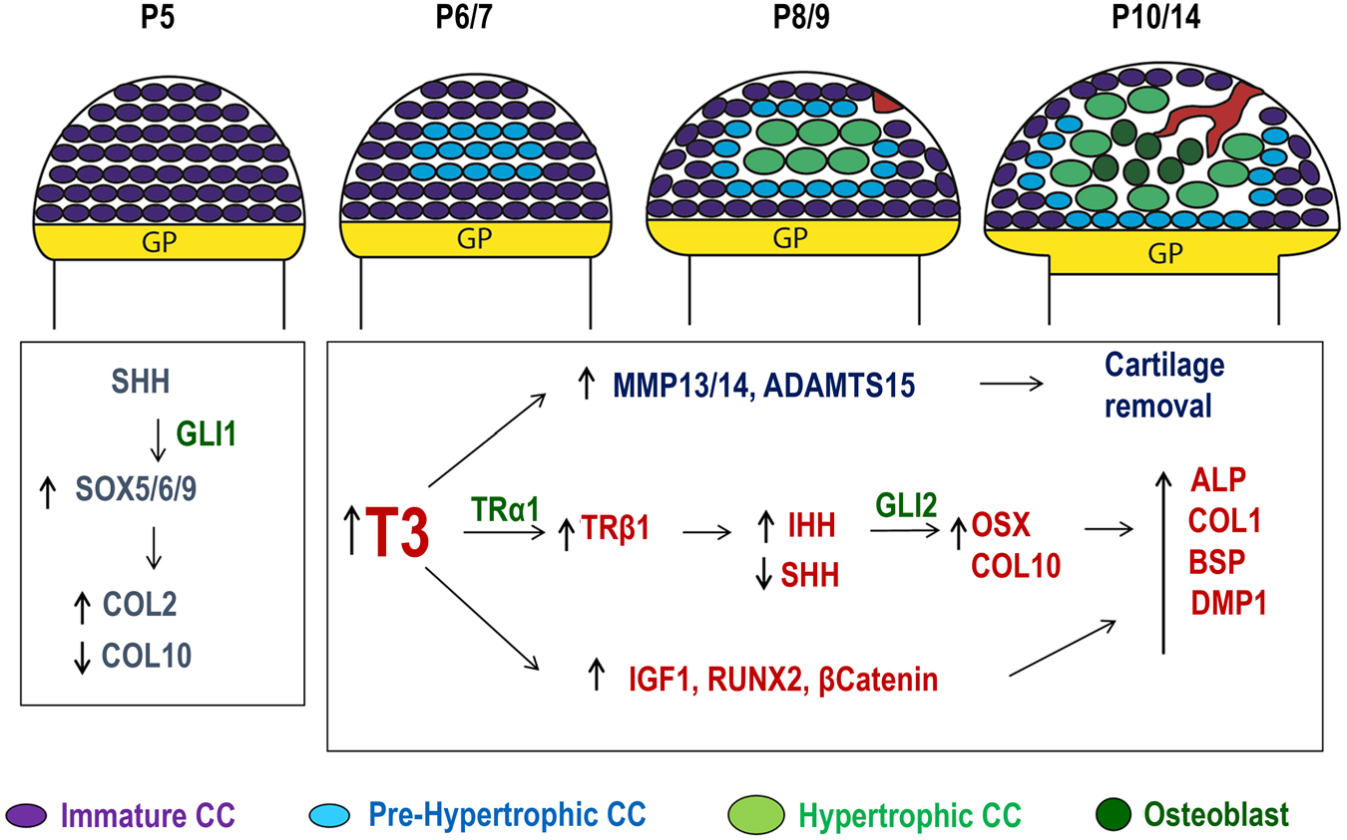
Model for early postnatal development of the SOC. During embryonic and early postnatal development when TH levels are low, epiphyseal chondrocytes express high levels of SHH which acts though Gli1 to maintain these cells in proliferative immature state by activating Sox5/6/9 transcriptional activity. At P6/P7, rise in TH increases THRβ1expression and thereby IHH expression. IHH acts through GLI2 to decrease SOX9 and COL2 expression, while MMP13 and ADAMTS5 expression increases to deplete the COL2 matrix. Pre-hypertrophic CCs begin to express COL10 and OSX in the P8/P9 period. These in turn activate DMP1 and ALP in the SOC, meanwhile blood vessels invade from the periphery of the articular CCs.

Taken together, these data identify hypertrophic chondrocytes as a novel source of bone forming cells at the epiphysis during secondary center ossification and confirms the importance of TH in its early regulation (Fig. 8). Based on our data and other published data, we propose a model in which the rise in systemic TH levels during the prepubertal growth period (day 5–14) acts through THRα1 to increase expression of THRβ1, which in turn increases expression of IHH and suppresses expression of SHH. IHH then acts through GLI2 to increase chondrocyte differentiation directed toward a hypertrophic fate. During TH deficiency states, THRβ1 expression is suppressed resulting from escape from THRβ1-mediated downregulation of SHH expression to inhibit chondrocyte differentiation by acting via GLI1 signaling. Thus, TH acts via THRα1 and THRβ1 to fine tune levels of SHH and IHH and, thereby, controls the transition of proliferating immature chondrocytes into mature hypertrophic chondrocytes in the epiphyses during secondary center ossification. The identification of molecular pathways for TH regulation of chondrocyte transdifferentiation into osteoblasts could provide opportunities for the development of novel targeted approaches to increase specific signaling to promote fracture healing in those instances where bones don’t heal normally.

## Materials and Methods

### Generation of mice

*ROSA-tdTomato* mice were crossed with the *Col2-Cre* ^*ERT2*^ transgenic mice. The offspring received an IP injection of 100 μg tamoxifen at post-natal day 3 (P3) and were euthanized at days 7, 8, 9, 10, 14 and week 12 to generate chondrocyte-specific lineage traces. Methimazole treated mice were generated via prenatal administration in the drinking water of the pregnant mothers^14^. *Tshr* ^*hyt/*+^ heterozygous mice with a point mutation in the coding region of the TSHR gene (*Tshr*) were purchased from the Jackson Laboratory (Bar Harbor, ME) (Table S1). Animals were obtained from locations itemized in the key resources table and housed in standard cages. For methimazole experiments, pregnant female mice were fed Kool-Aid water (without sugar added) containing 0.05% methimazole until pups were weaned. A sample size of two animals were used for the immunohistochemical experiments involving the *hyt/* + − P6-P10 experiments, the P8-P10 *Col2-Cre* ^*ERT2*^ *ROSA-tdTomato*, and methimazole treated *Col2-Cre* ^*ERT2*^ *ROSA-tdTomato* animals. The hourly P8-P9 *hyt/* + − animals had a sample size of 1, due to their time constraints. All injections were intraperitoneal, and performed at stated time points in the experimental methods. Animal tissue used was collected from the long bones/legs of sacrificed animals.

### Ethics Statement

Animals were housed according to the approved laboratory conditions in the animal facility unit at VA Loma Linda Healthcare System (Loma Linda, CA). All animal studies were performed on *mus musculus* under the consent and supervision of the Institutional Animal Care and Use Committee (IACUC) at the VA Loma Linda Healthcare System facility. All experimental procedures were evaluated and carried out in accordance with the protocols approved by the Institutional Animal Care and Use Committee of the VA Loma Linda Healthcare System. Isoflurane was used for anesthesia, and CO2 exposure was used for euthanasia followed by cervical dislocation. All procedures performed followed the ethical guidelines for animal studies.

### Immunohistochemistry

Parrafin or frozen tissues were sectioned and stained with primary antibodies. Consecutive tissue sections were deparaffinized in histochoice clearing agent (Sigma-Aldrich, St. Louis, MO), rehydrated in a graded series of ethanol and tap water solutions, and treated with 3% H_2_O_2_ for 30 minutes to inactivate endogenous peroxidase activity. The sections were then rinsed thoroughly with PBS (pH 7.4) and incubated in 2 mg/mL hyaluronidase (Sigma) (pH 7.4) for 30 minutes at 37 °C for epitope recovery. Each section was pretreated with a blocking solution containing normal goat serum for 20 minutes, and then incubated with primary antibodies for SOX9 (1:200), MMP13 (1:200), Caspase 3-active (1:40), SHH (1:200), DMP1 (1:200), COL1 (1:100), CD31 (1:200) (Novus Biologicals, Littleton, CO), OSX (1:300), EMCN (1:100), THRβ1 (1:500) and THRα1 (1:500) (Santa Cruz Biotechnology, inc., Dallas, TX), ALP (1:10), COL2 (1:10) (DSHB, Iowa City, IA), IHH (1:100), COL10 (1:100), or KI67 (1:100) (ABCAM, Cambridge, UK), BSPII (1:200) (gift from Dr. Renny T. Franceschi at University of Michigan School of Dentistry, Ann Arbor, Mi). After an overnight incubation at 4 °C, the sections were rinsed with PBS, and incubated with secondary anti-mouse, anti-rabbit fluorescent antibodies (Dylight 488 or 594) (Vector Laboratories, Burlingame, CA) or anti-rat alexa 488 (Invitrogen, Carlsbad, CA), and DAPI (Sigma). Colormetric IHC images were prepared with a Vectastain ABC Kit (Vector Laboratories, Burlingame, CA) (Table S1). Antibodies control stains were done in tandem and included secondary antibodies with no primary.

### Mouse epiphysis primary cell culture

Tibial epiphyses were surgically isolated from 7-day-old *Tshr* ^*hyt/*+^ or *Tshr* ^*hyt/hyt*^ and were incubated in serum-free α-MEM containing 0.5% BSA, 50 μg/ml ascorbic acid, 1 mM-glycerol phosphate, 100 U/ml penicillin, and 100 μg/ml streptomycin at 37 °C in humidified air with 5% CO_2_ for 24 hours. Cells were transduced with *Shh*-*OE* adenovirus as described^32, 33^. Cells were cultured for an additional 2 days followed by RNA extraction for real-time RT-PCR.

### Cell transduction

ATDC5 cells were maintained in DMEM/F12 medium containing 10% FBS, penicillin (100 units/ml), and streptomycin (100 μg/ml). For knockdown studies, ATDC5 cells were transduced with commercially available Lentiviral particles expressing shRNA against mouse *Thrβ1*, *Ihh*, or control GFP (Table S1) in 6-well culture plates in the presence of 8 μg/ml of polybrene, as described by the manufacturer. Twenty four hours later, the cells were cultured in fresh osteoblast differentiation medium and treated with T_3_ (10 ng/ml)/T_4_ or vehicle, followed by RNA extraction for real-time RT-PCR.

### RNA extraction and quantitative PCR

RNA was extracted from cultured cells as described in^34^. Epiphysis of long bones were isolated from *Tshr* ^*hyt/*+^ and *Tshr* ^*hyt/hyt*^ mice and ground to powder in liquid nitrogen using a mortar and pestle prior to RNA extraction. An aliquot of RNA (25 ng) was reverse transcribed with an oligo (dT)12–18 primer into cDNA in a 20 μL reaction volume. The real time PCR reaction contained 0.5 μL of template cDNA, 1X SYBR GREEN master mix (Thermo), and 100 nM of specific forward and reverse primers in a 25 μL reaction volume. Primers for peptidyl prolyl isomerase A (PPIA) were used to normalize the expression data for the genes of interest. The primer sequences used for real time PCR are listed in Table S2.

### Statistical Analysis

Data are presented as the mean ± standard error of means (SEM) from 4–6 mice from each group. Significant difference was determined as P ≤ 0.05 or P ≤ 0.01. Data were analyzed by Student’s t-test or two-way ANOVA as appropriate.

## Electronic supplementary material

Supplementary Tables and Figures
