# Supplementary material for: Epiphyseal bone formation occurs via thyroid hormone regulation of chondrocyte to osteoblast transdifferentiation
Source: Sci Rep. 2017 Sep 5;7:10432. doi: 10.1038/s41598-017-11050-1 (PMC5585223; doi:10.1038/s41598-017-11050-1)
Supplement: Supplementary file 1 — Supplementary Tables and Figures [file 41598_2017_11050_MOESM1_ESM.pdf]

Epiphyseal bone formation occurs via thyroid hormone regulation of chondrocyte to osteoblast transdifferentiation

Patrick Aghajanian[1], Weirong Xing[1,2], Shaohong Cheng[1], Subburaman Mohan[1,2,3,4*].

Author Affiliations

[1]Musculoskeletal Disease Center, Veterans Affairs Loma Linda Healthcare System, Loma Linda, California
[2]Department of Medicine, Loma Linda University, Loma Linda, California
[3]Department of Orthopedics, Loma Linda University, Loma Linda, California
[4]Department of Biochemistry, Loma Linda University, Loma Linda, California
[*]Subburaman.mohan@va.gov

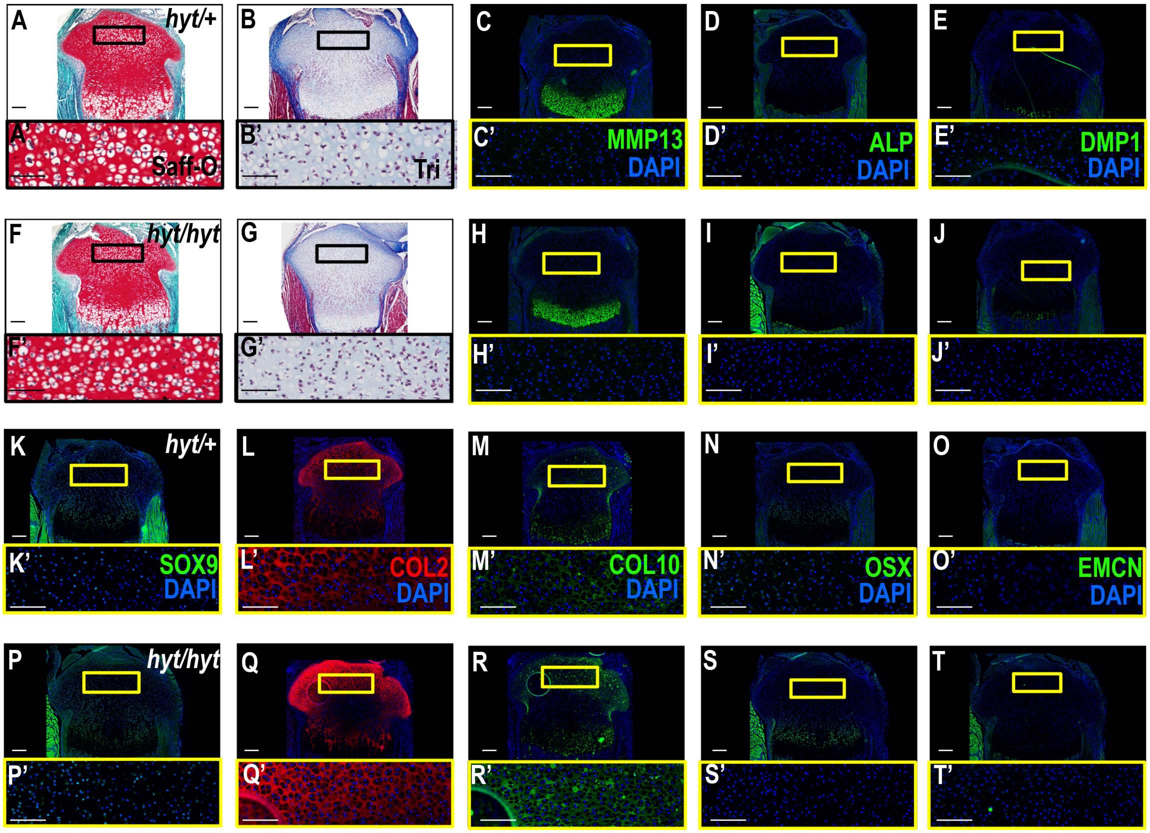

Figure S1. 6 Day IHC expression of chondrogenic and osteogenic factors in *hyt/hyt* and *hyt/+* animals.
(A-E', K-O') *Hyt/+* expression of P6 Safranin-O (A), trichrome (B), MMP13 (C). ALP (D). DMP1 (E). SOX9 (K), COL2 (L), Col10 (M), OSX (N), and EMCN (O).
(F-J', P-T') *Hyt/+* expression of P6 Safranin-O (F), trichrome (G), MMP13 (H). ALP (I). DMP1 (J). SOX9 (P), COL2 (Q), Col10 (R), OSX (S), and EMCN (T).
Yellow or black boxes enlarge related regions, " ' " denotes enlarged region (e.g., A').
Large Scale bars are 100 µm and small scale bars are 200 µm.

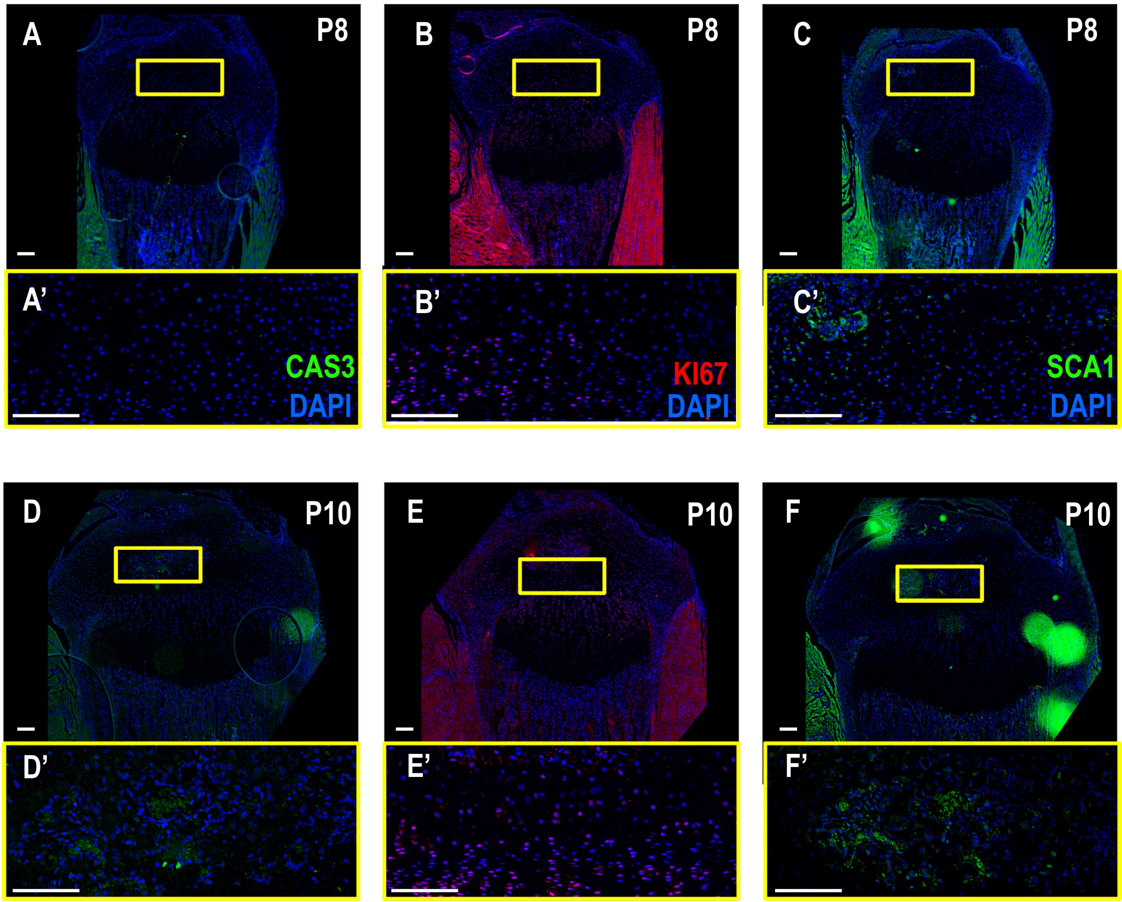

Figure S2. The presumptive SOC has limited/no apoptosis, proliferation or MSC invasion at initiation.
(A-C) P8, the center of the epiphysis does not express active CAS3 (A), KI67 (B), or SCA1 (C). Limited expression of some markers can be seen in the surrounding tissue. (D-F) P10, No Detectible CAS3 (D), very little expression of KI67 (E) and SCA1 (F) in the secondary center of ossification.
Yellow boxes enlarge related regions, "ʻ" denotes enlarged region (e.g., A'). "A" denotes significance. Large Scale bars are 100 µm and small scale bars are 200 µm.

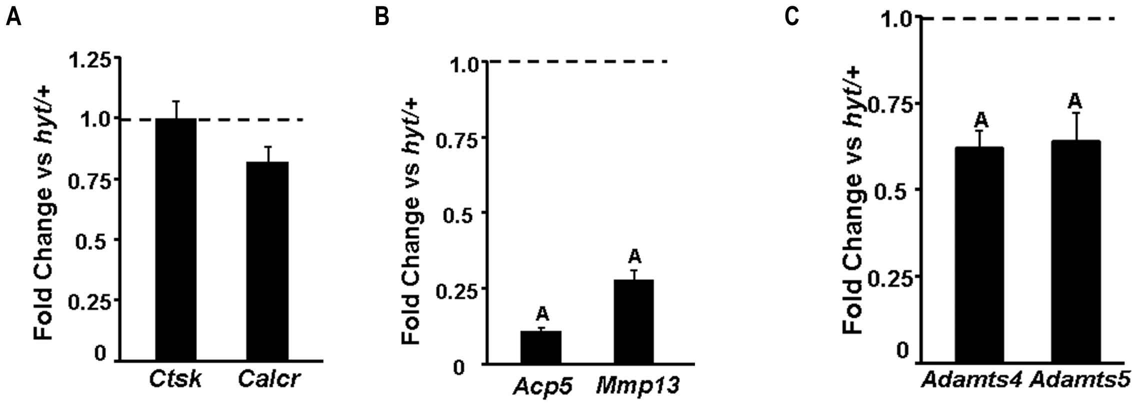

Figure S3. Matrix degradation is not related to osteoclast activity.
(A, B) P7 SOC CCs from *hyt/hyt* animals exhibited virtually no change in *Ctsk* or *Calcr* mRNA expression (A), while *Acp5* and *Mmp13* were reduced 9 and 3.7 fold, respectively when compared to *hyt/*+.
(C) P21 SOC CCs from *hyt/hyt* animals *Adamts4* and *Adamts5* mRNA expression were both reduced 1.6 fold when compared to *hyt/*+
All RNA data are represented as a mean ± SEM, “A” denotes significance.

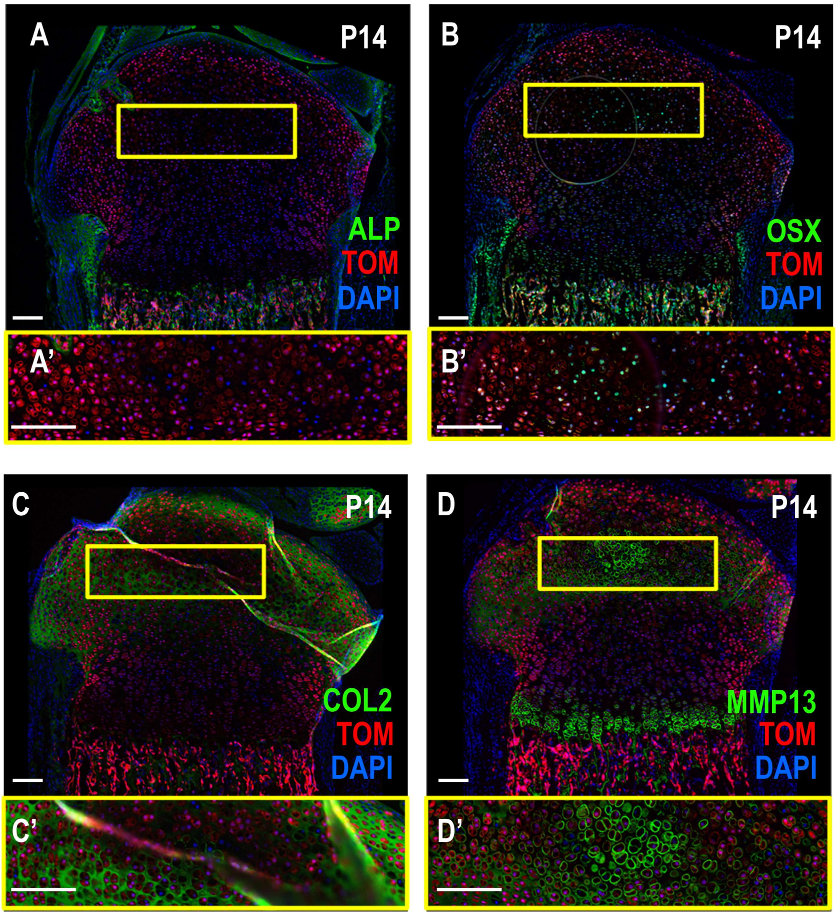

Figure S4. CCs do not transdifferentiate without thyroid hormone.
(A, B) At P14, epihyses express little to no bone formation factors such as ALP (A), though some TOM/OSX colabeling can be seen (B).
Yellow boxes enlarge related regions, " ' " denotes enlarged region (e.g., A'). Large Scale bars are 100 μm and small scale bars are 200 μm.
(C, D) P14 epiphyses are comprised mainly of COL2 immature CCs (C), but do form hypertrophic cells in the center as shown with MMP13 (D).

Table S1 Key Resources Table

REAGENT or RESOURCE	SOURCE	IDENTIFIER
**Antibodies**		
IHH	Abcam	Cat#ab39634
KI67	Abcam	Cat#ab66155
COL10	Abcam	Cat#ab58632
ALP	DSHB	Cat#B4-78
COL2	DSHB	Cat#CIIC1
SOX9	Novus Biologicals	Cat#NBP1-85551
MMP13	Novus Biologicals	Cat#NBP1-45723
Caspase 3-active	Novus Biologicals	Cat#AF835
SHH	Novus Biologicals	Cat#NBP2-22139
DMP1	Novus Biologicals	Cat#NBP1-89484
OSX	Santa Cruz Biotechnology	Cat#sc-22536-R
EMCN	Santa Cruz Biotechnology	Cat#sc-65495
THRβ1	Santa Cruz Biotechnology	Cat#sc-10822
THRα1	Santa Cruz Biotechnology	Cat#sc-10819
Dylight 488	Vector Labs	Cat#DI-1788, Cat#DI-2788
Dylight 594	Vector Labs	Cat#DI-2794, Cat#DI-1794
**Chemicals, Peptides, and Recombinant Proteins**		
L-Thyroxine (T_4)	Sigma	Cat#T1775
3, 3', 5-Triiodo-L-Thyronine (T_3)	Sigma	Cat#T2877
Methimazole	Sigma	Cat#M8506
Tamoxifen	Sigma	Cat#T5648
DAPI	Sigma	Cat#D9542
Hyaluronidase	Sigma	Cat#H6254
**Critical Commercial Assays**		
Vectastain ABC Kit	Vector Labs	Cat#PK-6100
Power SYBR Green PCR Master Mix	Thermo Fisher Scientific	Cat#4367659
**Experimental Models: Cell Lines**		
ATDC5	Abgent	#CL1016
Ad-293	Stratagene	Cat#240085
**Experimental Models: Organisms/Strains**		
ROSA-tdTomato	Jackson Laboratory	Stock #007909
Col2-Cre^{ERT2}	Jackson Laboratory	Stock #006774
CBy.RF-Tshr^{hyt}*/J*	Jackson Laboratory	Stock #000805
**Recombinant DNA**		
IHH shRNA	Sigma	TRCN0000031066
THRB shRNA	Sigma	TRCN0000027061
SHH Overepxression construct	Crystal Lab through the Zhang Lab	(Huang, et al., 2014; Sato, et al., 1999)
**Sequence-Based Reagents**		
For Primer list see Table S2		

Table S2. Primer sequences used for real time PCR

	Forward primer sequence	Reverse primer sequence
Ppia	5'-CCATGGCAAATGCTGGACCA-3'	5'-TCCTGGACCCAAAACGCTCC-3'
Acp5	5'-CACTCAGCTGTCCTGGCTCAA-3'	5'-CTGCAGGTTGTGGTCATGTCC-3'
Adamts4	5'-CAGTGCCCGATTCATCACTG-3'	5'-GAGTCAGGACCGAAGGTCAG-3'
AdamtsS5	5'-CGAAGAGCACTACGATGCAGC-3'	5'-GCATGGAGGCCATCATCTTCAAT-3'
Aggrecan	5'-GACCAGGAAGGGAGGAGTAG-3'	5'-CAGCCGAGAAATGACACC-3'
Bsp	5'-AACGGGTTTCAGCAGACAACC-3'	5'-TAAGCTCGGTAAGTGTCGCCA
Calcr	5'-CGGACTTTGACACAGCAGAA-3'	5'-CAGCAATCGACAAGGAGTGA-3'
Col2	5'-TGGCTTCCACTTCAGCTATG-3'	5'-AGGTAGGCGATGCTGTTCTT-3'
Ctsk	5'-GAACGAGAAAGCCCTGAAGAGA-3'	5'-TATCGAGTGCTTGCTTCCCTTC-3'
Gli1	5'-GCACCACATCAACAGTGAGC-3'	5'-GCGTCTTGAGGTTTTCAAGG-3'
Gli2	5-TACCTCAACCCTGTGGATGC-3'	5'-CTACCAGCGAGTTGGGAGAG-3'
Gli3	5'-ATTCCCGTAGCAGCTCTTCA-3'	5'-AGATCCTAAGCCGACAGCAA-3'
Ihh	5'-CCCCAACTACAATCCCGACATC-3'	5'-CGCCAGCAGTCCATACTTATTTCG-3'
Mmp13	5'-CATCCATCCCGTGACCTTAT-3'	5'-TCATAACCATTCAGAGCCCA-3'
Rankl	5'-GACTCCTGCAGGAGGATGAA-3'	5'-GTCCTCTTGGTACCACGATC-3'
Shh	5'-GCCTACAAGCAGTTTATTCCCA-3'	5'-GTGAGTTCCTTAAATCGTTCGG-3'
Sox9	5'-CGGAGGAAGTCGGTGAAGA-3'	5'-GTCGGTTTTGGGAGTGGTG-3'
Thrα1	5'-CTGCCTTGCGAAGACCAGATC-3'	5'-CAGCCTGCAGCAGAGCCACTTCCG-3'
Thrβ1	5'-GGTGCTGGATGACAGCAAGA-3'	5'-GCATTCACGATGGGTGCTTGT-3'

Table S2. Primer sequences used for real time PCR

List of primer sequences used in RT-PCR experiments. Abbreviations: Peptidylprolyl Isomerase A, *Ppia*; Acid Phosphatase 5, *Acp5*; ADAM Metallopeptidase With Thrombospondin Type 1 Motif 4, *Adamts4*; ADAM Metallopeptidase With Thrombospondin Type 1 Motif , *Adamts5*; Bone Sialoprotein, *Bsp*; Calcitonin Receptor; *Calcr*; Collagen Type 2, *Col2*; Cathepsin K, *Ctsk*; GLI Family Zinc Finger 1, *Gli1*; GLI Family Zinc Finger 2, *Gli2*; GLI Family Zinc Finger 3, *Gli3*; Indian Hedgehog, *Ihh*; Matrix Metalloprotease 13, *Mmp13*; Rank Ligand, *Rankl*; Sonic Hedgehog, *Shh*; SRY-Box 9, *Sox9*; *Thyroid Hormone Receptor Alpha 1, Thrα1* ; Thyroid Hormone Receptor Beta 1, *Thrβ1*.
